# Development and Content Validity of the Bilateral Vestibulopathy Questionnaire

**DOI:** 10.3389/fneur.2022.852048

**Published:** 2022-03-17

**Authors:** Lisa van Stiphout, Israt Hossein, Merel Kimman, Susan L. Whitney, Andrianna Ayiotis, Michael Strupp, Nils Guinand, Angélica Pérez Fornos, Josine Widdershoven, Ángel Ramos-Macías, Vincent Van Rompaey, Raymond van de Berg

**Affiliations:** ^1^Division of Balance Disorders, Department of Otorhinolaryngology and Head and Neck Surgery, Maastricht University Medical Center, School for Mental Health and Neuroscience, Maastricht, Netherlands; ^2^Department of Clinical Epidemiology and Medical Technology Assessment (KEMTA), Maastricht University Medical Center, Maastricht, Netherlands; ^3^Department of Physical Therapy, School of Health and Rehabilitation Sciences, University of Pittsburgh, Pittsburgh, PA, United States; ^4^Department of Otolaryngology–Head and Neck Surgery, Johns Hopkins University School of Medicine, Baltimore, MD, United States; ^5^Department of Biomedical Engineering, Johns Hopkins University School of Medicine, Baltimore, MD, United States; ^6^Department of Neurology, German Center for Vertigo, Ludwig-Maximilians University, Munich, Germany; ^7^Service of Otorhinolaryngology Head and Neck Surgery, Department of Clinical Neurosciences, Geneva University Hospitals, Geneva, Switzerland; ^8^Department of Otorhinolaryngology and Head and Neck Surgery, Faculty of Medicine and Health Sciences, Antwerp University Hospital, University of Antwerp, Antwerp, Belgium; ^9^Department of Otolaryngology, Head and Neck Surgery, Complejo Hospitalario Universitario Insular Materno Infantil de Gran Canaria, Las Palmas, Spain

**Keywords:** bilateral vestibulopathy, Patient Reported Outcome Measure (PROM), questionnaire, vestibular impairment, symptoms bilateral vestibulopathy

## Abstract

**Background:**

To date, the burden and severity of the full spectrum of bilateral vestibulopathy (BVP) symptoms has not yet been measured in a standardized manner. Since therapeutic interventions aiming to improve BVP symptoms are emerging, the need for a new standardized assessment tool that encompasses the specific aspects of BVP arises. Therefore, the aim of this study was to develop a multi-item Patient Reported Outcome Measure (PROM) that captures the clinically important symptoms of BVP and assesses its impact on daily life.

**Methods:**

The development of the Bilateral Vestibulopathy Questionnaire (BVQ) consisted of two phases: (I) initial item generation and (II) face and content validity testing. Items were derived from a literature review and individual semi-structured interviews focusing on the full spectrum of reported BVP symptoms (I). Subsequently (IIa), individual patient interviews were conducted using “thinking aloud” and concurrent verbal probing techniques to assess the comprehensibility of the instructions, questions and response options, and the relevance, missing domains, or missing items. Interviews continued until saturation of input was reached. Finally, international experts with experience in the field of the physical, emotional, and cognitive symptoms of BVP participated in an online focus group to assess the relevance and comprehensiveness of the BVQ (IIb).

**Results:**

The BVQ consisted of two sections. The first section included 50 items scored on a six-point Likert scale arranged into seven constructs (i.e., imbalance, oscillopsia, other physical symptoms, cognitive symptoms, emotional symptoms, limitations and behavioral changes and social life). The second section consisted of four items, scored on a visual analog scale from 0 to 100, to inquire about limitations in daily life, perceived health and expectations regarding future recovery. Interviews with BVP patients [*n* = 8, 50% female, mean age 56 years (range 24–88 years)] and the expert meeting confirmed face and content validity of the developed BVQ.

**Conclusion:**

The BVQ, which was developed to assess the spectrum of BVP symptoms and its impact on daily life, proved to have good face and content validity. It can be used to characterize current self-reported symptoms and disability and to evaluate symptom burden before and after therapeutic interventions in future research and clinical practice.

## Introduction

Bilateral vestibulopathy (BVP) is a chronic disorder which is defined by bilateral loss or reduction of vestibular function due to deficits of the vestibular organs, vestibular nerves, the brain, or a combination of the above (1, 2). The leading symptoms of BVP include oscillopsia during walking or quick head/body movements (movement-induced blurred vision), unsteadiness when walking or standing and worsening of unsteadiness on uneven ground or in darkness (2). Previous literature demonstrated that BVP also results in cognitive or emotional complaints (3–6). In particular, an association between BVP and symptoms such as sadness, fear, anger, difficulties with dual tasking and spatial anxiety was described (3–11). This ultimately results in behavioral changes such as avoiding activities or performing activities more slowly and with greater attention (6). Together these symptoms negatively impact daily functioning and quality of life and increase socio-economic burden (12–15).

BVP can pose a diagnostic challenge since it is a heterogeneous disorder with multiple identified etiologies and with various clinical characteristics due to different involvement within the vestibular system (16–24). Currently, BVP is diagnosed using the criteria from the Classification Committee of the Bárány Society, which include symptoms of oscillopsia and imbalance, together with a reduced vestibular function measured with caloric testing and/or video head impulse test and/or torsion swing test (2). Since the current BVP criteria were developed as a diagnostic tool, they primarily focus on physical complaints combined with measuring vestibular reflexes and not on the full spectrum of BVP symptoms (i.e., it is not designed to portray the burden of disease in patients with BVP). Additionally, earlier research has demonstrated that the extent of the perceived dizziness related handicap is often not correlated with results from vestibular reflex tests (25, 26). Therefore, evaluating the burden of disease cannot be accomplished by focusing on the diagnostic criteria alone.

Generic questionnaires such as the Short-Form Health Survey (SF-36), the Health-Utilities-Index (HUI) and EuroQol-5D-5L (EQ-5D-5L) but also more specific questionnaires such as the Dizziness Handicap Inventory (DHI) and Hospital Anxiety and Depression Scale (HADS) have been used to characterize symptoms, quality of life and emotional health in BVP populations (6, 12). However, due to the generic nature of these questionnaires, they are evidently not able to accurately capture the full spectrum and the specific aspects of BVP symptoms relevant to patients (6, 27).

To date, the burden and severity of the full spectrum of BVP symptoms are not measured in a standardized way. Since therapeutic interventions focusing on improving BVP symptoms are emerging (e.g., the vestibular implant, balance belt and noisy galvanic stimulation), the need for a standardized assessment tool that embraces the specific aspects of BVP arises (28–39). Thorough evaluation of BVP symptoms and disease burden via patient-relevant and disease-specific parameters before and after therapeutic interventions is also crucial for measuring the effectiveness of these interventions (40, 41). A targeted questionnaire has the potential to improve clinical decision making as it enables clinicians and patients to establish realistic and shared treatment goals, taking patient preferences into account (42–45).

Consequently, the development of a multi-item Patient Reported Outcome Measure (PROM), that captures the clinically important and patient-relevant symptoms of the BVP population would be of value. The aim of this study was to develop a PROM to assess the spectrum of BVP symptoms and its impact on daily life. The development and face and content validity testing of the Bilateral Vestibulopathy Questionnaire (BVQ) are described in this article.

## Conceptual Model and Context

**Figure 1:**
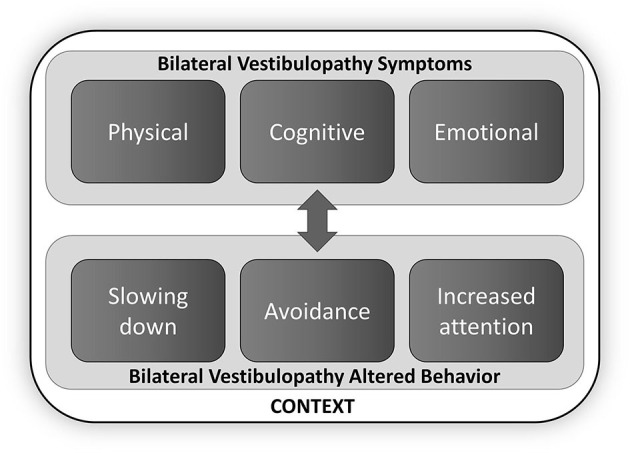
Conceptual framework of the Bilateral Vestibulopathy Questionnaire (BVQ) [adapted from Lucieer et al. (6)]. The spectrum of BVP symptoms is categorized in three different domains (the physical, cognitive, and emotional domain), which can lead to context-specific behavioral changes. Double arrow indicates the interrelation between concepts.

Results from a qualitative study assessing symptoms of patients with BVP were used as foundation for the conceptual framework of the BVQ (6, 46). This conceptual framework is centered around the full spectrum of BVP symptoms and categorizes them in three different domains: the physical, cognitive, and emotional domain. The burden of these symptoms increases when performing certain activities such as cycling or driving a car, leading to limitations in carrying out these activities (47). Consequently, patients show altered behavior such as slowing down or paying more attention while performing activities or even avoiding specific activities (6). Since BVP symptoms and BVP patients' behavior are interrelated, the conceptual model embodies all three domains of BVP symptoms and BVP behavioral concepts [adapted from the model of altered behavior due to BVP when performing activities by Lucieer et al. (6), Figure 1].

The BVQ was developed for use in patients with BVP. It was developed for evaluative applications to characterize the current self-reported symptoms and disability and to depict symptom burden before and after therapeutic interventions (41). Furthermore, the questionnaire is aimed to be used as an addition to the diagnostic BVP criteria to portray symptom severity, for patient counseling, and shared clinical decision making in a research and clinical setting (2, 42–45).

## Methods

The development of the BVQ consisted of two phases: (I) initial item generation and (II) face and content validity testing. Phase II included cognitive interviews to gain insight in patients' perspectives (IIa) and an international expert meeting for obtaining input from several BVP experts (IIb). All phases of development are in agreement with the COSMIN guideline for PROM development (48).

### Initial Item Generation

The initial items for the BVQ were formulated by a panel of experts consisting of two otorhinolaryngologists with an expertise in Vestibular Medicine, a vestibular researcher with a focus on BVP and an expert in health outcome research and PROM development. The themes and items were derived from a previous literature review and individual semi-structured patient interviews focusing on the full spectrum of reported symptoms of BVP (5, 6). A detailed procedure regarding the systematic review and qualitative data analysis was described by Lucieer et al. (5, 6). Discussions with the panel of experts were used to define the first longlist of relevant items for inclusion in the questionnaire, the questionnaire instructions, the recall period, the response options and lay-out. This first version was formulated in Dutch. The questionnaire was translated to English, in consultation with a native English speaker, and sent to another group of experts located in Europe and the United States (two otorhinolaryngologists with an expertise in Vestibular Medicine, one neurologist with an expertise in Vestibular Medicine, one neural prosthesis research specialist with an expertise in vestibular implantation, one physical therapist focusing on balance disorders and one vestibular researcher with a focus on BVP and vestibular implantation). Open feedback was asked on the initial items, different types of response options, the questionnaire instructions and recall period. Feedback of all experts was incorporated to remodel the first longlist version for face and content validity testing. The version for face and content validity testing was translated from English back to Dutch. The first version of the BVQ is presented in the result section of this report (phase I).

### Face and Content Validity Test

#### Cognitive Patient Interviews

Individual patient interviews (*n* = 8) were conducted to assess the comprehensibility of the instructions, questions and response options, and the relevance, missing domains or missing items. Patients with BVP diagnosed at the Maastricht University Medical Center+ according to the diagnostic criteria of the Bárány Society were asked to participate in this study (2). Inclusion criteria for BVP included imbalance and/or oscillopsia during walking or head movements, a reduced bithermal caloric response (sum of bithermal maximal peak slow phase velocity bilaterally <6°/s), and/or a reduced vestibular-ocular-reflex (VOR) gain as measured by horizontal vHIT (bilateral VOR gain < 0.6) and/or torsion swing test (VOR gain < 0.1). It was required that patients were able to speak the Dutch language and to understand written Dutch. Interviews were conducted individually, either face-to-face or virtually, depending on the patient's preference, and continued until saturation of input was reached. Saturation was reached when interviews no longer provided new information in addition to previous interviews.

Interviews were conducted in Dutch following a semi-structured interview guide and consisted of two phases (Supplementary Data Sheet 1). At first, an open discussion took place in which patients were instructed to define what items should be included in a BVP questionnaire. During the second phase, the BVQ was provided to patients and they were asked to read the instruction, questions, and their answers aloud [i.e., a think-aloud technique was used (49)]. Patients were asked to verbalize their thoughts while answering the questions. If patients were unclear or were hesitating in formulating their thoughts or answers, the interviewer probed further into the response to gain additional information about the interpretation and understanding of the items, construct, or overall questionnaire. The interviewer used both spontaneous and pre-scripted probes during the interview. The interviews were moderated and facilitated by experienced researchers with medical backgrounds and trained in interviewing patients and qualitative data analysis (LvS and IH). Neither the interviewer nor the observer had established patient relationships prior to conducting this study. All interviews were recorded and transcribed verbatim and the transcripts were anonymized. The transcripts were subjected to manual qualitative analysis by two data coders (LvS and IH). Coding was carried out by highlighting quotations or phrases in the text and assigning each quotation or phrase a code representing predefined categories. Consensus meetings were organized between the two researchers to identify (in)consistencies in their findings and to confirm whether the interpretations were correct. All codes were summarized in a spreadsheet database, which was used for evaluating whether to delete, alter, add, or preserve items from the questionnaire. The methods, findings, analysis and interpretations of the interviews were reported in accordance with the consolidated criteria for reporting qualitative research (COREQ; Supplementary Data Sheet 2) (50).

Based on the results from the first set of cognitive interviews (*n* = 4), revisions were made to the questionnaire items and instructions during two consecutive panel expert meetings (LvS, IH, MK, RvdB). To confirm face and content validity of the revised items, further cognitive interviews were conducted. Based on the results from the final set of interviews (*n* = 4), some minor revisions were made to the questionnaire during a panel expert meeting to facilitate patient understanding of the items. Results from all interviews were combined and presented as the results from phase II of this report.

#### Expert Meeting

International experts with extensive experience in the field of the physical, emotional, and cognitive symptoms of BVP and mechanism of altered behavior due to BVP were invited to participate in an online focus group to assess the relevance and comprehensiveness of the BVQ. The meeting was led by two trained moderators (RvdB and LvS). Five experts (one otorhinolaryngologist with an expertise in Vestibular Medicine, one neurologist with an expertise in Vestibular Medicine, one physical therapist focusing on balance disorders and two vestibular researchers with a focus on BVP and vestibular implantation) discussed items that were selected during initial item generation (phase I) and the cognitive interviews (phase IIa) via a semi-structured interview format (Supplementary Data Sheet 3). The English version of the BVQ was used, due to the participation of international experts. Each item was discussed with respect to its relevance in clinical practice and the overall questionnaire was commented on whether it included all the relevant constructs and items of BVP (comprehensiveness). During this meeting, the final set of questions was selected in consensus. The meeting was recorded and transcribed verbatim and the transcripts were anonymized. The transcript was subjected to manual qualitative analysis by two data coders (LvS and IH) and data analysis was performed as described for the cognitive patient interviews (phase IIa). This phase led to the final version of the BVQ, which was sent to the patient group from phase IIa to confirm comprehensibility of any changes suggested by the experts.

### Ethical Considerations

This study was conducted in accordance with the legislation and ethical standards on human experimentation in the Netherlands and in accordance with the Declaration of Helsinki (amended version 2013). The medical ethical committee of Maastricht UMC+ approved this study (METC 2020-2215) and written informed consent was obtained from all patients participating in this study.

## Results

### Initial Item Generation

Items elicited from the systematic literature review, the individual patient interviews, the panel of experts' discussions and the online survey to six international experts resulted in a longlist consisting of two sections. The first section included seven constructs with a total of 44 items scored on a six-point (Likert-type) scale: three constructs regarding physical symptoms (imbalance, oscillopsia and other physical symptoms), one construct encompassing cognitive symptoms, one construct encompassing emotional symptoms, one construct comprising limitations and behavioral changes, and one construct regarding social life. A six-point Likert scale was chosen, since literature showed that patients were less likely to choose a neutral option out of convenience and could therefore provide answers with a higher discrimination compared to a five-point Likert scale (51). The 6-point Likert-scale included the following points: “never,” “rarely,” “sometimes,” “regularly,” “frequently,” and “always.” Six items in the behavioral construct included a “not applicable” answer option since these items could not be answered by some patients in specific cases (e.g., not being able to drive a car yourself when not having a driver's license). The second part consisted of three scale items, scored on a visual analog scale from 0 to 100, to inquire about limitations in daily life (two items) and perceived health at the moment of completing the questionnaire. Both sections contained short instructions. It was emphasized that the items were focused on the symptoms patients have experienced due to BVP. Patients were encouraged to choose the answer that best suited their situation. The chosen recall period was one week since BVP symptoms can vary per day, depending on the patient's daily activities. Furthermore, a recall period of one week was considered to be most suitable for evaluative assessments after therapy. A shorter recall period may cause over- or underestimation of symptoms and a longer recall period could increase the risk of recall bias (52, 53).

An overview of the content and items constructed during this phase is shown in Supplementary Data Sheet 4 Table 1.

### Face and Content Validity Test

#### Cognitive Patient Interviews

Eight BVP patients [(50% female, mean age 56 years (range 24–88 years)] were approached to participate in this study and completed the initial round of individual interviews. Included BVP etiologies were idiopathic (*n* = 2), DFNA9 (*n* = 2), Cogan's syndrome (*n* = 1), head trauma (*n* = 1), ototoxicity due to gentamicin treatment (*n* = 1), and meningitis (*n* = 1). All included patients fulfilled the Bárány Society Criteria and had no comorbidities. The mean bithermal caloric response was 1.3°/s and 1.5°/s for the right and left ear respectively. The mean VOR gain was 0.1 measured with torsion swing test and 0.22 bilaterally measured with horizontal vHIT.

Two rounds of individual cognitive interviews were organized with four participants included in each round. All interviews lasted between 75 and 120 min with an average duration of 93 min. The first round of interviews consisted of four online video-recorded meetings [mean age 36 years (range 24–53 years)]. The relatively young age included in the first round was due to the online nature of the first round of interviews during the COVID-19 pandemic, since younger participants were more willing and able to participate online. Elderly participants were more comfortable with face-to-face interviews.

From the first round of interviews some issues were identified regarding the formulation of items or missing items that required alteration of the BVQ. Based on feedback from the first set of interviews, revisions were made to the questionnaire items and instructions during a panel expert meeting (LvS, IH, MK, RvdB). To confirm face and content validity of the (revised) BVQ, a second round of interviews was conducted. This round consisted of four face-to-face interviews [mean age 77 years (range 68–88 years)] and resulted in some minor adjustments.

Results from the interviews indicated that all constructs were considered relevant and overall that the questions were clear and concise. Some minor textual changes were required to increase comprehensibility. For example, some patients mentioned that the items about “falling” and “tripping” were similar in phrasing. Therefore, the formulation of the item about “tripping” was changed to “trip without falling” (construct imbalance). The word “dizziness” was changed to “lightheadedness” since some patients experienced difficulty with the word “dizziness” in combination with standing up fast (construct other physical symptoms). Next to this, patients reported wanting to make a comparison with their situation before their diagnosis of BVP regarding the items “forgetfulness” and “concentration” (construct cognition). Lastly, to increase the comprehensibility and ease of reading, the words “limit” and “avoid” were underlined (construct limitations and behavioral changes) and the order of the scale questions was altered to create a more logical sequence.

The interviews pointed out that items were missing regarding imbalance when changing positions (construct imbalance), problems with multitasking (construct cognition), and emotions such as sadness, embarrassment, and loneliness (construct emotion). An item regarding the potential positive influence of BVP on social interactions was difficult or unpleasant to answer according to the patients and was therefore removed from the questionnaire.

All patients were able to answer the questions on a six-point Likert scale and appreciated the differences between steps on the scale from “never” to “always.” The “not applicable” response option for some items was found relevant. However, two patients mentioned that the “not applicable” answer option was not clearly visible when completing the questionnaire. Therefore, after the first round of cognitive interviews an instruction was added above this construct to point out this extra answer option.

All instructions were clear to patients and sufficiently concise. However, some patients did not relate all their symptoms to having BVP and answered some questions with “never” although they clearly expressed experiencing these symptoms. Therefore, minor textual changes were made to the introduction after the first round of interviews to increase clarity. No changes in recall period were made since the majority of the patients pointed out that one week was an appropriate recall period for the questionnaire.

The second round of interviews indicated that no more substantial changes were needed, and face and content validity were considered good. Overall, the interviews pointed out that patients felt that the questionnaire covered all relevant aspects of BVP and that it made them feel heard. The length of the questionnaire was not experienced as bothersome. Instead, patients clearly expressed that the length of the questionnaire made them feel that they were taken seriously. Finally, after inquiring, all patients indicated that the COVID-19 pandemic did not affect the completion of the questionnaire.

An overview of the content and items altered during this phase is shown in Supplementary Data Sheet 4 Table 2.

#### Expert Meeting

The validity of the BVQ was discussed with regard to the relevance and comprehensiveness with international experts with extensive experience in the field of BVP during a 90 min online expert meeting. In general, experts advised to make minor textual changes which resulted in shorter and more concise items (e.g., “I feel tired” instead of “I feel tired due to my symptoms”). These alterations made the questionnaire more concise. Three items were added to make the questionnaire complete: one item regarding oscillopsia while walking (construct oscillopsia), one item regarding difficulties with multitasking while walking (construct cognition), and one scale question regarding expectations concerning future recovery. No changes were made to the instructions, recall period or response options and no items were removed from the questionnaire.

To verify that the comprehensibility of the questionnaire remained unchanged after incorporating the alterations from the expert meeting and to test all items in their final form, the questionnaire was discussed with five patients [40% female, mean age 65 years (range 39–88 years)] from phase IIa. All five patients confirmed comprehensibility and relevance of all items and the comprehensiveness of the overall questionnaire. The final instrument included 54 items (i.e., 50 items scored on a six-point Likert scale and four VAS items) which is shown in Supplementary Data Sheet 4 Table 3.

## Discussion

This paper describes the development and face and content validity testing of the Bilateral Vestibulopathy Questionnaire (BVQ), a PROM to assess the spectrum of BVP symptoms and their impact on functioning and daily life. Face and content validity tests with eight BVP patients and five international experts confirmed the relevance, comprehensibility, and comprehensiveness of the developed BVQ, which included 50 items arranged into seven constructs (imbalance, oscillopsia, other physical symptoms, cognitive symptoms, emotional symptoms, limitations and behavioral changes and social life) and four scale items.

Specific PROMs, such as the BVQ, can be used in clinical practice to [1] improve communication between patients and health care professionals, [2] facilitate personalized care and shared clinical decision making and [3] improve patient satisfaction (54–59). Moreover, in research and clinical settings, regular assessment of patients' symptoms and functioning with PROMs can be useful for obtaining information about patients' experiences regarding their disorder and symptom evolution, and for monitoring and evaluating treatment effectiveness (54–56). The latter is especially important when introducing novel treatment strategies such as the vestibular implant. By administering the BVQ before and after a new intervention, it can be used complementary to vestibular reflex testing as a valuable outcome measure for evaluating treatment efficacy over time in future research and clinical practice.

To evaluate quality of life, symptom burden and emotional health in BVP populations both generic and specific questionnaires were used previously (6, 12). One of the main advantages of generic questionnaires is the large applicability. In other words, generic questionnaires can be administered in various patient populations and as a result, they can be valuable for comparison of symptoms or quality of life between different patient populations (60, 61). They are however less sensitive to small changes and may include domains which are not applicable for every patient population. This is also seen in the BVP population for generic questionnaires such as the EQ-5D-5L and the HUI3, as previous literature illustrated that these questionnaires did not fully capture BVP symptom burden (5). Firstly, this can be explained by the items included in generic questionnaires. For example, domains such as dexterity (HUI3) and pain (HUI3 and EQ-5D-5L) are less relevant symptoms in the BVP population. Next to this, the HUI3 does not distinguish between static or dynamic visual acuity (domain vision). The latter, which is an important symptom in the BVP population (oscillopsia) and has a major impact on quality of life, is not measured accurately in this way. Secondly, previous literature showed that chronically changed situations can alter patients views of what is considered normal, a phenomenon called adaptation (62). It is hypothesized that this also applies for the BVP population. Consequently, the HUI3 and EQ-5D-5L overall index scores would not give an accurate representation of BVP symptom burden and quality of life. To overcome this issue, the BVQ includes specific aspects of BVP symptoms and contexts relevant to patients.

It is stated that more specific questionnaires such as the DHI and HADS have greater sensitivity for small changes in health state or symptom burden, but at the same time have the disadvantage that they mainly focus on only one aspect of health or quality of life (63). However, the symptoms experienced by BVP patients are much broader than the items included in the DHI and HADS (6). As a result, the use of these questionnaires alone can overlook many aspects important to patients living with BVP. The BVQ includes the full extent of BVP symptoms and is therefore expected to provide a better representation of the perceived symptom burden and to be more sensitive to disease-specific changes. Subsequently, the BVQ should be more suitable for evaluation of treatment effectiveness.

A limitation of this study was the relatively small number of participants for the individual patient interviews (*n* = 8) and expert meeting (*n* = 5). However, since interviews continued until data saturation was reached, the number of BVP patients included for face and content validity testing was adequate (48, 50). Moreover, no overlap between patients included in phase I (initial item generation) and phase IIa (face and content validity testing) was ensured. Furthermore, the expert meeting was held internationally, to ensure good quality feedback from well-known experts in the field of BVP. Additionally, a minor risk of sampling bias could not be prevented since patients willing to participate in this study were highly motivated and willing to contribute to the development of a PROM for BVP. Therefore, possibly patients with stronger opinions and/or severe symptoms and disease burden were overrepresented in this study. To minimize the risk of sampling bias, a variety of patients were selected with differences in age, symptom duration, sex, educational backgrounds, and etiologies.

Future work on the BVQ includes testing of the construct validity and reliability using quantitative methods in a large study population. After assessment of all psychometric properties, the BVQ will be translated into different languages followed by cross-cultural validation to facilitate its use internationally.

## Conclusion

The BVQ, which was developed to assess the spectrum of BVP symptoms and its impact on daily life, proved to have good face and content validity. It can be used to characterize current self-reported symptoms and disability and to depict symptom burden before and after therapeutic interventions.

## Data Availability Statement

The raw data supporting the conclusions of this article will be made available by the authors, without undue reservation.

## Ethics Statement

This study was conducted in accordance with the legislation and ethical standards on human experimentation in the Netherlands and in accordance with the Declaration of Helsinki (amended version 2013). The Medical Ethical Committee of Maastricht UMC+ approved this study (METC 2020-2215) and written informed consent was obtained from all patients participating in this study.

## Author Contributions

LS, MK, and RB designed the study. LS wrote the manuscript. LS, RB, MK, SW, AA, Á-RM, MS, and VV contributed to developing the first longlist. LS and IH performed cognitive patient interviews and conducted the analysis. LS and RB conducted the expert meeting. MK and RB supervised the writing and edited the manuscript. SW, MS, NG, AP, JW, Á-RM, and VV reviewed the manuscript. All authors contributed to the article and approved the submitted version.

## Conflict of Interest

LS was supported through funding of MED-EL (Innsbruck, Austria). RB, AP, and NG received funding for travel from MED-EL. MS is Joint Chief Editor of the Journal of Neurology, Editor in Chief of Frontiers of Neuro-otology and Section Editor of F1000 and has received speaker's honoraria from Abbott, Auris Medical, Biogen, Eisai, Grünenthal, GSK, Henning Pharma, Interacoustics, J&J, MSD, NeuroUpdate, Otometrics, Pierre-Fabre, TEVA, UCB, and Viatris. MS also receives support for clinical studies from Decibel, U.S.A., Cure within Reach, U.S.A. and Heel, Germany and acts as a consultant for Abbott, AurisMedical, Heel, IntraBio and Sensorion. SW is a paid consultant for Intelligent Automation (a BlueHalo Company). The funders had no role in study design, data collection, data analysis, interpretation of data, decision to publish, or preparation of the manuscript. The remaining authors declare that the research was conducted in the absence of any commercial or financial relationships that could be construed as a potential conflict of interest.

## Publisher's Note

All claims expressed in this article are solely those of the authors and do not necessarily represent those of their affiliated organizations, or those of the publisher, the editors and the reviewers. Any product that may be evaluated in this article, or claim that may be made by its manufacturer, is not guaranteed or endorsed by the publisher.
