# Supplementary material for: Development and Content Validity of the Bilateral Vestibulopathy Questionnaire
Source: Front Neurol. 2022 Mar 17;13:852048. doi: 10.3389/fneur.2022.852048 (PMC8968143; doi:10.3389/fneur.2022.852048)
Supplement: Supplementary file 1 [file Data_Sheet_1.PDF]

Supplementary Material 1 - Interview Guide patient interviews

Characteristics of the interview

- Face-to-face individual interviews or online individual interviews
- Location: Participants home (face-to-face or online)
- Semi-structured interviews (main question with potential sub-questions, respondent driven topics)
- Planned duration: 60-90 min
- Digitally audio-recorded
- Language: Dutch

Aim of the interview

- To assess the comprehensibility of the instructions, questions and response options (face validity), and the relevance and missing domains or missing items (content validity).

Main questions and potential sub-questions

1. Brief introduction:
   - Introduce moderator and observer.
   - Explain the purpose of the cognitive interview.
   - Could you please briefly introduce yourself?
   - When were you diagnosed with bilateral vestibulopathy?
   - How do you experience living with bilateral vestibulopathy?
2. Phase 1 (open discussion): Inquire about the main topic
   - Participants are asked to name items/topics that they believe are essential for an assessment tool that aims to gain insight into the symptoms and complaints associated with bilateral vestibulopathy.
     "What questions do you think should be asked in this questionnaire?"
     "Please define what items should be included in a BV questionnaire."

Handing out questionnaires.

3. Phase 2: Assessment of the pilot questionnaire
   - Participants are asked to read the instructions, the questions and to answers the questions aloud. Next to this, patients are asked to verbalize their thoughts while answering the questions. If patients are unclear during their thinking aloud or are hesitating in formulating their thoughts or answers, the interviewer probes further into the response (using follow-up questions) to gain additional information about the interpretation and understanding of the items, construct or overall questionnaire. The interviewer is allowed to use both spontaneous and pre-scripted probes during the interview. Examples:

Instructions:
"Are the instructions clearly described?"
"Would you word the instructions in a different way?"

Key questions per item
For each item in the questionnaire, the interviewer asks the participant additional questions. Some examples are mentioned below;
"Do you think this question is relevant?"
"Do you think the answer options are logical / appropriate for this question?"
"Do you think the answer options are relevant?"
"Is this question clearly defined?"
"Would you word the questions differently? If so, how?"
"To what extent is it difficult to answer this question? Why is that?"
"How did you arrive at this answer? Why not higher or lower?"
"What makes you hesitant to answer the question?"

Key questions per construct
For each construct, the interviewer asks the participant additional questions. Some examples are mentioned below;
"Are there certain questions that you are missing below this section? If so, which ones and why?"
"Are there any questions that you consider unnecessary in this section? If so, which ones and why?"

Recall period
"To what extent did you find the recall period 'last week' applicable?"
"Would you choose a different recall period?"

Overall
"What did you think of the structure of the questionnaire/did you think that the questionnaire has a logical structure?"
"Did you feel that this questionnaire (all questions taken together) covered your complaints well?"
"Are there any important topics missing from this questionnaire?"
"Were there any questions that you found difficult to answer?"
"Did you understand what was meant by all the questions?"
"Are there important points that have not yet been addressed?"
"Were the complaints different before the COVID quarantine started in March? If so, how?"
"Did the current COVID situation impact the way you filled in the questionnaire?"

Summary/conclusion

Give a compact summary of the interview.

- "Are there any important items/things that we have not discussed?"

Turn off recording equipment, check if all files are recorded and saved. Mention again for what the results will be used:

- "The results of this interview will be used to improve the current bilateral vestibulopathy questionnaire."
- "I would like to remind you that this report will be anonymous."
- "Thank you for participating, the interview has now ended."
