# Supplementary material for: Development and Content Validity of the Bilateral Vestibulopathy Questionnaire
Source: Front Neurol. 2022 Mar 17;13:852048. doi: 10.3389/fneur.2022.852048 (PMC8968143; doi:10.3389/fneur.2022.852048)
Supplement: Supplementary file 2 [file Data_Sheet_2.PDF]

frontiers

Supplementary Material 2 – Consolidated criteria for reporting qualitative studies (COREQ): 32-item checklist

Developed from:

Tong A, Sainsbury P, Craig J. Consolidated criteria for reporting qualitative research (COREQ): a 32-item checklist for interviews and focus groups. International Journal for Quality in Health Care. 2007. Volume 19, Number 6: pp. 349 – 357

**No. Item**	**Guide questions/description**	**Reported on Page/Section**
**Domain 1: Research team and reflexivity**		
Personal Characteristics		
1. Interviewer/facilitator	Which author/s conducted the interview or focus group?	Title page & Methods section
2. Credentials	What were the researcher's credentials? E.g. PhD, MD	Title page
3. Occupation	What was their occupation at the time of the study?	Title page & Methods section
4. Gender	Was the researcher male or female?	Title page
5. Experience and training	What experience or training did the researcher have?	Title page
Relationship with participants		
6. Relationship established	Was a relationship established prior to study commencement?	Methods section
7. Participant knowledge of the interviewer	What did the participants know about the researcher? e.g. personal goals, reasons for doing the research	Methods section
8. Interviewer characteristics	What characteristics were reported about the interviewer/facilitator? e.g. Bias, assumptions, reasons and interests in the research topic	Methods section

No. Item	Guide questions/description	Reported on Page (section)
**Domain 2: study design**		
Theoretical framework		
9. Methodological orientation and Theory	What methodological orientation was stated to underpin the study? e.g. grounded theory, discourse analysis, ethnography, phenomenology, content analysis	Title page & Methods section
Participant selection		
10. Sampling	How were participants selected? e.g. purposive, convenience, consecutive, snowball	Methods section
11. Method of approach	How were participants approached? e.g. face-to-face, telephone, mail, email	Methods section
12. Sample size	How many participants were in the study?	Results section
13. Non-participation	How many people refused to participate or dropped out? Reasons?	Methods section
Setting		
14. Setting of data collection	Where was the data collected? e.g. home, clinic, workplace	Methods section
15. Presence of non-participants	Was anyone else present besides the participants and researchers?	Methods section
16. Description of sample	What are the important characteristics of the sample? e.g. demographic data, date	Results section
Data collection		
17. Interview guide	Were questions, prompts, guides provided by the authors? Was it pilot tested?	Methods section & Supplementary Material
18. Repeat interviews	Were repeat inter views carried out? If yes, how many?	No (inferred from Methods section)
19. Audio/visual recording	Did the research use audio or visual recording to collect the data?	Methods section

20. Field notes	Were field notes made during and/or after the inter view or focus group?	Methods section
21. Duration	What was the duration of the inter views or focus group?	Methods section
22. Data saturation	Was data saturation discussed?	Methods section
23. Transcripts returned	Were transcripts returned to participants for comment and/or correction?	No

**No. Item**	**Guide questions/description**	**Reported on Page (section)**
**Domain 3: analysis and findings**		
Data analysis		
24. Number of data coders	How many data coders coded the data?	Methods section
25. Description of the coding tree	Did authors provide a description of the coding tree?	Methods section
26. Derivation of themes	Were themes identified in advance or derived from the data?	Methods section
27. Software	What software, if applicable, was used to manage the data?	Methods section
28. Participant checking	Did participants provide feedback on the findings?	Yes, Method & Result section
Reporting		
29. Quotations presented	Were participant quotations presented to illustrate the themes/findings? Was each quotation identified? e.g. participant number	No
30. Data and findings consistent	Was there consistency between the data presented and the findings?	Yes, there was.<br>(Results & Discussion section)
31. Clarity of major themes	Were major themes clearly presented in the findings?	Yes, they were.<br>(Results & Discussion section)
32. Clarity of minor themes	Is there a description of diverse cases or discussion of minor themes?	(Results & Discussion section)
