# Supplementary material for: Development and Content Validity of the Bilateral Vestibulopathy Questionnaire
Source: Front Neurol. 2022 Mar 17;13:852048. doi: 10.3389/fneur.2022.852048 (PMC8968143; doi:10.3389/fneur.2022.852048)
Supplement: Supplementary file 3 [file Data_Sheet_3.PDF]

Supplementary Material 3 – Interview Guide Expert meeting

Characteristics of the interview

- Online Videorecord Focus group
- Location: online
- Semi-structured interviews (main question with potential sub-questions, respondent driven topics)
- Planned duration: 90 min
- Digitally audio-recorded
- Language: English

Aim of the interview

- To assess the relevance and comprehensiveness of the BVQ.

Main questions and potential sub-questions

1. Brief introduction:
   - Could you please briefly introduce yourself?
2. Inquire about the main topic after showing the questionnaire online:
   - Experts will be asked to read the instructions and questions individually. Afterwards, each section and question will be discussed. The moderator will ensure adequate group discussion and make sure everyone gets the same amount of time to speak. Examples:

     Instruction and recall period
     "Is the recall period of one week relevant?"
     "Do you think another recall period is more relevant?"

     Key questions per item
     For each question of the questionnaire, the interviewer will ask the experts additional questions. "Do you think this question is relevant?"
     "Do you think the answer options are logical / suitable for this question?"

     Key questions per construct
     For each construct, the interviewer will ask the experts additional questions.
     "Are there certain questions missing regarding this construct? If yes, which question and why?"
     "Are there certain questions that are unnecessary? If yes, which question and why?"

The overall questionnaire

"What do think of the structure of the questionnaire/do you think the questionnaire has a logical structure?"
"Do you think the overall questionnaire is comprehensive?"
"Do you think there are any important questions missing in this questionnaire (including all questions)?"

Summary/conclusion

Give a compact summary of the focus group session.

- "Are there any important items/things that we have not discussed?"

Turn off recording equipment, check if all files are recorded and saved. Mention again for what the results will be used:

- "The results of this focus group session will be used to improve the current bilateral vestibulopathy questionnaire."
- "I would like to remind you that this report will be anonymous."
- "Thank you for participating, the focus group session has now ended."
