# Supplementary material for: Development and Content Validity of the Bilateral Vestibulopathy Questionnaire
Source: Front Neurol. 2022 Mar 17;13:852048. doi: 10.3389/fneur.2022.852048 (PMC8968143; doi:10.3389/fneur.2022.852048)
Supplement: Supplementary file 4 [file Data_Sheet_4.PDF]

Supplementary Material 4 – Bilateral Vestibulopathy Questionnaire

Table 1. Set of items for the BVQ PROM development after the initial item generation phase.

**Bilateral Vestibulopathy Questionnaire – item generation phase**		
Instruction<br>This questionnaire is about the symptoms you have experienced **during the past week** due to your vestibular loss.<br>Read the questions carefully and check the appropriate box behind each question.<br>When in doubt, check the answer that best suits your situation.		
Imbalance		Answer scale
1	I experience imbalance and/or feel drunk during daily activities.	*1 (never) – 6 (always)*
2	I experience imbalance and/or feel drunk when walking on uneven surfaces (like in the woods, at the beach or in the snow).	*1 (never) – 6 (always)*
3	I experience imbalance and/or feel drunk when walking in the dark (such as walking in a room with dimmed lights).	*1 (never) – 6 (always)*
4	When walking, I need to pay attention to the ground to avoid falling.	*1 (never) – 6 (always)*
5	I trip more often than before I had vestibular loss.	*1 (never) – 6 (always)*
6	I fall more often than before I had vestibular loss.	*1 (never) – 6 (always)*
Oscillopsia		
7	I have blurred vision while walking.	*1 (never) – 6 (always)*
8	I have blurred vision while being in a train, bus, or car on a bumpy road.	*1 (never) – 6 (always)*
9	When walking, I have to stand still to recognize faces or to read (road) signs.	*1 (never) – 6 (always)*
10	I have blurred vision while chewing on my food.	*1 (never) – 6 (always)*
Other psychical symptoms		
11	I experience difficulties with fast head movements, like turning my head to the right or left when crossing the street.	*1 (never) – 6 (always)*
12	I get dizzy when standing up too fast.	*1 (never) – 6 (always)*
13	I feel tired due to my symptoms.	*1 (never) – 6 (always)*
Cognition		
14	I must pay close attention to my balance when walking or turning my head.	*1 (never) – 6 (always)*
15	I find it difficult to orient myself in new places, like in new cities or buildings.	*1 (never) – 6 (always)*
16	I find it difficult to estimate distances, like the depth of a staircase or distance to oncoming traffic.	*1 (never) – 6 (always)*
17	I am forgetful.	*1 (never) – 6 (always)*
18	I find it difficult to concentrate well.	*1 (never) – 6 (always)*
Emotion		
19	I generally feel well.	*1 (never) – 6 (always)*
20	I worry a lot.	*1 (never) – 6 (always)*

21	I generally feel confident when performing daily activities.	*1 (never) – 6 (always)*
22	I am afraid to fall.	*1 (never) – 6 (always)*
23	I feel in control.	*1 (never) – 6 (always)*
24	I am bothered by fast movements surrounding me, like traffic flashing by.	*1 (never) – 6 (always)*
25	I am bothered by environments with loud sounds, such as a concert or a construction site.	*1 (never) – 6 (always)*
26	I am bothered by busy environments surrounding me, such as a busy supermarket or a shopping mall.	*1 (never) – 6 (always)*
27	I generally don't feel confident when performing daily activities.	*1 (never) – 6 (always)*
Behavior and limitations		
28	I feel limited in daily life activities due to my symptoms.	*1 (never) – 6 (always)*
29	I avoid daily life activities due to my symptoms.	*1 (never) – 6 (always)*
30	I feel limited when performing vigorous activities, such as sports like running, due to my symptoms.	*1 (never) – 6 (always) – N/A*
31	I avoid performing vigorous activities, such as sports like running, due to my symptoms.	*1 (never) – 6 (always) – N/A*
32	I feel limited when performing moderate activities, such as gardening or playing golf due to my symptoms.	*1 (never) – 6 (always) – N/A*
33	I avoid performing moderate activities, such as gardening or playing golf due to my symptoms	*1 (never) – 6 (always) – N/A*
34	I feel limited in travelling due to my symptoms.	*1 (never) – 6 (always)*
35	I avoid travelling due to my symptoms	*1 (never) – 6 (always)*
36	I feel limited when driving a car myself due to my symptoms.	*1 (never) – 6 (always) – N/A*
37	I avoid driving a car myself due to my symptoms.	*1 (never) – 6 (always) – N/A*
38	I feel limited in my social life activities due to my symptoms.	*1 (never) – 6 (always)*
39	I avoid social life activities due to my symptoms.	*1 (never) – 6 (always)*
40	I need to perform daily activities at a slower pace due to my symptoms.	*1 (never) – 6 (always)*
Social life		
41	My symptoms negatively affect my close relationships.	*1 (never) – 6 (always)*
42	I feel like people fail to understand my situation.	*1 (never) – 6 (always)*
43	I depend on other people to perform daily activities.	*1 (never) – 6 (always)*
44	My symptoms positively affect my close relationships.	*1 (never) – 6 (always)*
VAS scale questions		
45	How limited do you feel in daily life?<br>The scale below is numbered from 0 to 100.<br>0 means that you are not limited in daily life due to your symptoms.<br>100 means that you are extremely limited in daily life due to your symptoms.<br>Mark an X on the scale to indicate how much you feel limited in daily life.	*Scale 0 (not limited in daily life) – 100 (extremely limited in daily life)*
46a	What is the most important symptom, due to your vestibular loss, you would like to have improved?<br>Please fill in your answer here:	*Open answer*

46b	How much do you suffer from this symptom (see the answer you filled in at question 46a)?<br>The scale below is numbered from 0 to 100.<br>0 means you do not suffer from this symptom.<br>100 means you extremely suffer from this symptom.<br>Mark an X on the scale to indicate how much you suffer from this symptom.	*Scale 0 (no suffering) – 100 (extreme suffering)*
47	How do you feel today?<br>The scale below is numbered from 0 to 100.<br>0 means you cannot feel worse than how you are feeling today.<br>100 means you cannot feel better than how you are feeling today.<br>Please note: the scale below is marked from ‘extremely bad’ (0) to ‘extremely good’ (100).<br>Mark an X on the scale to indicate how you feel today.	*Scale 0 (extremely bad) – 100 (extremely good)*

The items are developed in Dutch language and developed for the Dutch population. These items were translated into English for the purpose of this publication. An officially validated version in English will be made.

Table 2. Set of items for the BVQ PROM development after 2 rounds of cognitive interviews (n=8).

**Bilateral Vestibulopathy Questionnaire – Changes after cognitive interviews (n=8)**		
Instruction<br>This questionnaire is about the symptoms you have experienced **during the past week** due to your vestibular loss.<br>Complete this questionnaire as applicable to YOUR situation. There are no right or wrong answers. Please select only <u>one</u> answer. When in doubt, check the answer that best suits your situation.		
Imbalance		Answer scale
1	I experience imbalance and/or feel drunk during daily activities.	*1 (never) – 6 (always)*
2	I experience imbalance and/or feel drunk when walking on uneven surfaces (like in the woods, at the beach or in the snow).	*1 (never) – 6 (always)*
3	I experience imbalance and/or feel drunk when walking in the dark (such as walking in a room with dimmed lights).	*1 (never) – 6 (always)*
4	When walking, I need to pay attention to the ground to avoid falling.	*1 (never) – 6 (always)*
5	I fall more often than before I had vestibular loss.	*1 (never) – 6 (always)*
6	I trip more often (without falling) than before I had vestibular loss.	*1 (never) – 6 (always)*
7	I experience imbalance while switching positions (such as crouching, bending down, or standing up).	*1 (never) – 6 (always)*
Oscillopsia		
8	I have blurred vision while walking.	*1 (never) – 6 (always)*
9	I have blurred vision while being in a train, bus, or car on a bumpy road.	*1 (never) – 6 (always)*
10	When walking, I have to stand still to recognize faces or to read (road) signs.	*1 (never) – 6 (always)*
11	I have blurred vision while chewing on my food.	*1 (never) – 6 (always)*
Other psychical symptoms		
12	I experience difficulties with fast head movements, like turning my head to the right or left when crossing the street.	*1 (never) – 6 (always)*
13	I experience light-headedness when standing up too fast.	*1 (never) – 6 (always)*
14	I feel tired due to my symptoms.	*1 (never) – 6 (always)*
Cognition		
15	I must pay close attention to my balance when walking or turning my head.	*1 (never) – 6 (always)*
16	I find it difficult to orient myself in new places, like in new cities or buildings.	*1 (never) – 6 (always)*
17	I find it difficult to estimate distances, like the depth of a staircase or the distance to oncoming traffic.	*1 (never) – 6 (always)*
18	I am more forgetful than before I had vestibular loss.	*1 (never) – 6 (always)*
19	I find it more difficult to concentrate well than before I had vestibular loss.	*1 (never) – 6 (always)*
20	I experience difficulties with multitasking (such as walking while talking on the phone).	*1 (never) – 6 (always)*
Emotion		
21	I generally feel cheerful.	*1 (never) – 6 (always)*

22	I worry a lot.	*1 (never) – 6 (always)*
23	I generally feel confident when performing daily activities.	*1 (never) – 6 (always)*
24	I feel lonely.	*1 (never) – 6 (always)*
25	I am afraid to fall.	*1 (never) – 6 (always)*
26	I feel in control over my life.	*1 (never) – 6 (always)*
27	I am bothered by fast movements surrounding me, like traffic flashing by.	*1 (never) – 6 (always)*
28	I am bothered by environments with loud sounds, such as a concert or a construction site.	*1 (never) – 6 (always)*
29	I am bothered by busy environments surrounding me, such as a busy supermarket or a shopping mall.	*1 (never) – 6 (always)*
30	I generally don't feel confident when performing daily activities.	*1 (never) – 6 (always)*
31	I generally feel sad.	*1 (never) – 6 (always)*
32	I am embarrassed by my balance problems.	*1 (never) – 6 (always)*
Behavior and limitations		
Instruction<br>*Some of the questions below have an additional answer option: "not applicable" (N/A). Only check this box if the question does not apply to you.*		
33	I feel limited in daily life activities due to my symptoms.	*1 (never) – 6 (always)*
34	I avoid daily life activities due to my symptoms.	*1 (never) – 6 (always)*
35	I feel limited when performing vigorous activities, such as sports like running, due to my symptoms.	*1 (never) – 6 (always) – N/A*
36	I avoid performing vigorous activities, such as sports like running, due to my symptoms.	*1 (never) – 6 (always) – N/A*
37	I feel limited when performing moderate activities, such as gardening or playing golf due to my symptoms.	*1 (never) – 6 (always) – N/A*
38	I avoid performing moderate activities, such as gardening or playing golf due to my symptoms	*1 (never) – 6 (always) – N/A*
39	I feel limited in travelling due to my symptoms.	*1 (never) – 6 (always)*
40	I avoid travelling due to my symptoms	*1 (never) – 6 (always)*
41	I feel limited when driving a car myself due to my symptoms.	*1 (never) – 6 (always) – N/A*
42	I avoid driving a car myself due to my symptoms.	*1 (never) – 6 (always) – N/A*
43	I feel limited in my social life activities due to my symptoms.	*1 (never) – 6 (always)*
44	I avoid social life activities due to my symptoms.	*1 (never) – 6 (always)*
45	I need to perform daily activities at a slower pace due to my symptoms.	*1 (never) – 6 (always)*
Social life		
46	My symptoms negatively affect my close relationships.	*1 (never) – 6 (always)*
47	I feel like people fail to understand my situation.	*1 (never) – 6 (always)*
48	I depend on other people to perform daily activities.	*1 (never) – 6 (always)*
~~49~~	My symptoms positively affect my close relationships.	*1 (never) – 6 (always)*
VAS scale questions		
49	How limited do you feel in daily life?<br>The scale below is numbered from 0 to 100.<br>0 means that you are not limited in daily life due to your symptoms.	*Scale 0 (not limited in daily life) – 100 (extremely limited in daily life)*

	100 means that you are extremely limited in daily life due to your symptoms.<br>Mark an X on the scale to indicate how much you feel limited in daily life.	
50a	What is the most important symptom, due to your vestibular loss, you would like to have improved?<br>Please fill in your answer here:	*Open answer*
50b	How much do you suffer from this symptom (see the answer you filled in at question 50a)?<br>The scale below is numbered from 0 to 100.<br>0 means you do not suffer from this symptom.<br>100 means you extremely suffer from this symptom.<br>Mark an X on the scale to indicate how much you suffer from this symptom.	*Scale 0 (no suffering) – 100 (extreme suffering)*
51	How do you feel today?<br>The scale below is numbered from 0 to 100.<br>0 means you cannot feel worse than how you are feeling today.<br>100 means you cannot feel better than how you are feeling today.<br>Please note: the scale below is marked from ‘extremely bad’ (0) to ‘extremely good’ (100).<br>Mark an X on the scale to indicate how you feel today.	*Scale 0 (extremely bad) – 100 (extremely good)*

Green color indicates minor textual changes or the addition of a new item.
Red color indicates that an item/sentence/word is removed.

The items are developed in Dutch language and developed for the Dutch population. These items were translated into English for the purpose of this publication. An officially validated version in English will be made.

Table 3. Set of items for the BVQ PROM development after the expert meeting.

**Bilateral Vestibulopathy Questionnaire – Changes after expert meeting (n=5)**		
Instruction This questionnaire is about the symptoms you have experienced **during the past week** due to your balance problems. Read the questions carefully and check the appropriate box behind each question. Complete this questionnaire as applicable to YOUR situation. There are no right or wrong answers. Please select only one answer. When in doubt, check the answer that best suits your situation.		
Imbalance		Answer scale
1	I experience imbalance and/or feel drunk during daily activities.	*1 (never) – 6 (always)*
2	I experience imbalance and/or feel drunk when walking on uneven surfaces (like in the woods, at the beach or in the snow).	*1 (never) – 6 (always)*
3	I experience imbalance and/or feel drunk when walking in reduced light.	*1 (never) – 6 (always)*
4	When walking, I need to pay attention to the ground to avoid falling.	*1 (never) – 6 (always)*
5	I have fallen.	*1 (never) – 6 (always)*
6	I have come close to falling.	*1 (never) – 6 (always)*
7	I experience imbalance while changing positions (such as crouching, bending down, reaching or standing up).	*1 (never) – 6 (always)*
Oscillopsia		
8	I have blurred vision while walking.	*1 (never) – 6 (always)*
9	I have blurred vision while travelling (such as being in a train, bus, car or on a bike).	*1 (never) – 6 (always)*
10	When walking, I have to stand still to recognize faces or to read (road) signs.	*1 (never) – 6 (always)*
11	I have blurred vision while chewing on my food.	*1 (never) – 6 (always)*
12	I have blurred vision when quickly turning my head.	*1 (never) – 6 (always)*
Other psychical symptoms		
13	I experience difficulties with fast head movements, like turning my head to the right or left when crossing the street.	*1 (never) – 6 (always)*
14	I experience light-headedness when standing up fast.	*1 (never) – 6 (always)*
15	I feel tired due to my symptoms.	*1 (never) – 6 (always)*
Cognition		
16	I must pay close attention to my balance when walking or turning my head.	*1 (never) – 6 (always)*
17	I easily get lost in new places.	*1 (never) – 6 (always)*
18	I find it difficult to judge distances, like the distance to oncoming traffic.	*1 (never) – 6 (always)*
19	I am more forgetful than before I had vestibular loss.	*1 (never) – 6 (always)*
20	I find it more difficult to concentrate well than before I had vestibular loss.	*1 (never) – 6 (always)*
21	I experience difficulties with doing more than one thing at a time.	*1 (never) – 6 (always)*
22	I experience difficulties with doing things while walking.	*1 (never) – 6 (always)*
Emotion		
23	I generally feel happy.	*1 (never) – 6 (always)*

24	I worry a lot.	*1 (never) – 6 (always)*
25	When performing daily activities, I generally feel confident.	*1 (never) – 6 (always)*
26	I feel lonely.	*1 (never) – 6 (always)*
27	I am afraid to fall.	*1 (never) – 6 (always)*
28	I feel I have control over my life.	*1 (never) – 6 (always)*
29	I am bothered by fast movements surrounding me, like traffic flashing by.	*1 (never) – 6 (always)*
30	I am bothered by noisy environments, such as a concert or a construction site.	*1 (never) – 6 (always)*
31	I am bothered by busy environments surrounding me, such as a busy supermarket or a shopping mall.	*1 (never) – 6 (always)*
32	I generally don't feel confident when performing daily activities.	*1 (never) – 6 (always)*
33	I generally feel sad.	*1 (never) – 6 (always)*
34	I am embarrassed by my balance problems.	*1 (never) – 6 (always)*
Behavior and limitations		
Instruction<br>*Some of the questions below have an additional answer option: "not applicable" (N/A). Only check this box if the question does not apply to you.*		
35	I feel limited in daily life activities due to my symptoms.	*1 (never) – 6 (always)*
36	I avoid daily life activities due to my symptoms.	*1 (never) – 6 (always)*
37	I feel limited when performing vigorous activities, such as sports like running, due to my symptoms.	*1 (never) – 6 (always) – N/A*
38	I avoid performing vigorous activities, such as sports like running, due to my symptoms.	*1 (never) – 6 (always) – N/A*
39	I feel limited when performing moderate activities, such as gardening or playing golf due to my symptoms.	*1 (never) – 6 (always)*
40	I avoid performing moderate activities, such as gardening or playing golf due to my symptoms.	*1 (never) – 6 (always)*
41	I feel limited in travelling due to my symptoms.	*1 (never) – 6 (always)*
42	I avoid travelling due to my symptoms.	*1 (never) – 6 (always)*
43	I feel limited when driving a car myself due to my symptoms.	*1 (never) – 6 (always) – N/A*
44	I avoid driving a car myself due to my symptoms.	*1 (never) – 6 (always) – N/A*
45	I feel limited in my social activities due to my symptoms.	*1 (never) – 6 (always)*
46	I avoid social activities due to my symptoms.	*1 (never) – 6 (always)*
47	I need to perform daily activities slower due to my symptoms.	*1 (never) – 6 (always)*
Social life		
48	My symptoms negatively affect my close relationships.	*1 (never) – 6 (always)*
49	People don't understand my problem.	*1 (never) – 6 (always)*
50	I need the help of other people to perform daily activities.	*1 (never) – 6 (always)*
VAS scale questions		
51	How limited do you feel in daily life?<br>The scale below is numbered from 0 to 100.<br>0 means that you are not limited in daily life due to your symptoms.<br>100 means that you are extremely limited in daily life due to your symptoms.	*Scale 0 (not limited in daily life) – 100 (extremely limited in daily life)*

	Mark an X on the scale to indicate how much you feel limited in daily life.	
52a	What is the most important problem, due to your vestibular loss, you would like to have improved?<br>Please fill in your answer here:	*Open answer*
52b	How much does this problem affect your life (see the answer you filled in at question 52a)?<br>The scale below is numbered from 0 to 100.<br>0 means the problem does not affect your life.<br>100 means the problem affects your life extremely.<br>Mark an X on the scale to indicate how much you suffer from this symptom.	*Scale 0 (no suffering) – 100 (extreme suffering)*
53	How hopeful are you that you will get better?<br>The scale below is numbered from 0 to 100.<br>0 means you are not hopeful that you will get better.<br>100 means you are extremely hopeful that you will get better.<br>Mark an X on the scale to indicate how hopeful you are that you will get better.	*Scale 0 (not hopeful) – 100 (extremely hopeful)*
54	How would you rate your health today?<br>The scale below is numbered from 0 to 100.<br>0 means your health is extremely bad.<br>100 means your health is extremely good.<br>Please note: the scale below is marked from ‘extremely bad’ (0) to ‘extremely good’ (100).<br>Mark an X on the scale to indicate how you feel today.	*Scale 0 (extremely bad) – 100 (extremely good)*

Green color indicates minor textual changes or the addition of a new item.
Red color indicates that an item/sentence/word is removed.

The items are developed in Dutch language and developed for the Dutch population. These items were translated into English for the purpose of this publication. An officially validated version in English will be made.
